# Gene expression in the phenotypically plastic Arctic charr (*Salvelinus alpinus*): A focus on growth and ossification at early stages of development

**DOI:** 10.1111/ede.12275

**Published:** 2018-11-26

**Authors:** Samantha V. Beck, Katja Räsänen, Ehsan P. Ahi, Bjarni K. Kristjánsson, Skúli Skúlason, Zophonías O. Jónsson, Camille A. Leblanc

**Affiliations:** ^1^ Department of Aquaculture and Fish Biology Hólar University College Háskólinn á Hólum Sauðárkrókur Iceland; ^2^ Institute of Life‐ and Environmental Sciences University of Iceland Reykjavík Iceland; ^3^ Department of Aquatic Ecology, Eawag Swiss Federal Institute of Aquatic Science and Technology Dübendorf Switzerland; ^4^ Institute of Integrative Biology ETH Zürich Zürich Switzerland; ^5^ Institute of Zoology University of Graz Universitätsplatz 2 Graz Austria

## Abstract

Gene expression during development shapes the phenotypes of individuals. Although embryonic gene expression can have lasting effects on developmental trajectories, few studies consider the role of maternal effects, such as egg size, on gene expression. Using qPCR, we characterize relative expression of 14 growth and/or skeletal promoting genes across embryonic development in Arctic charr (*Salvelinus alpinus*). We test to what extent their relative expression is correlated with egg size and size at early life‐stages within the study population. We predict smaller individuals to have higher expression of growth and skeletal promoting genes, due to less maternal resources (i.e., yolk) and prioritization of energy toward ossification. We found expression levels to vary across developmental stages and only three genes (*Mmp9*, *Star*, and *Sgk1*) correlated with individual size at a given developmental stage. Contrary to our hypothesis, expression of *Mmp9* and *Star* showed a non‐linear relationship with size (at post fertilization and hatching, respectively), whilst *Sgk1* was higher in larger embryos at hatching. Interestingly, these genes are also associated with craniofacial divergence of Arctic charr morphs. Our results indicate that early life‐stage variation in gene expression, concomitant to maternal effects, can influence developmental plasticity and potentially the evolution of resource polymorphism in fishes.

## 1 INTRODUCTION

The ability of individuals to respond to environmental heterogeneity largely depends upon changes in gene expression, framed by physiological, behavioral, and anatomic constraints (Taugbøl, Arntsen, Østbye, & Vøllestad, 2014). Such interactions between genes and environment are most often realized through development, thus providing phenotypic variation upon which natural selection can act (e.g. West‐Eberhard, 2005).

During early stages of development, an organism's phenotype tends to be malleable (Hoverman & Relyea, 2007). The effects of such developmental plasticity often persist through to the adult phenotype (Gabriel, 2006; Godfrey, Lillycrop, Burdge, Gluckman, & Hanson, 2007; Holoch & Moazed, 2015) and may facilitate rapid evolutionary changes at the population level (Fusco & Minelli, 2010; Price, Qvarnstrom, & Irwin, 2003). One way to further our understanding of divergence of developmental trajectories is to examine the underlying changes in gene expression and deducing the related molecular processes (Politis et al., 2017; Schneider & Meyer, 2017). Examining patterns of gene expression across developmental stages can therefore provide a powerful approach to unveiling mechanisms involved in phenotypic diversification, and ultimately its role in evolution (Aubin‐Horth & Renn, 2009; Schneider, Li, Meyer, & Gunter, 2014).

Maternal effects, here defined as the causal influence of the maternal genotype or phenotype on offspring phenotype and fitness (Wolf & Wade, 2009), reflect a form of transgenerational effects (Kuijper & Hoyle, 2015; Uller, 2008). Maternal effects can have a strong impact on phenotypic development (Badyaev, 2008; Mousseau & Fox, 1998) and influence evolution at ecological time scales (see Räsänen & Kruuk, 2007; Uller, 2008). In many organisms, maternal allocation of resources (i.e., size and composition of propagules) is an important determinant of development at early life‐stages (Bernardo, 1996; Mousseau & Fox, 1998).

In fish, egg size is not only heritable (Kinnison, Unwin, Hendry, & Quinn, 2001; Su, Liljedahl, & Gall, 1997), but can vary both within and among females and is also influenced by the environment the mother experiences, as well as maternal age and size (Chambers & Leggett, 1996; Cogliati, Unrein, Stewart, Schreck, & Noakes, 2018; Leblanc, Benhaïm, Hansen, Kristjánsson, & Skúlason, 2011; Segers & Taborsky, 2012). In turn, egg size can strongly affect behavior and fitness of offspring (Benhaïm, Skúlason, & Hansen, 2003; Leblanc et al., 2011; Leblanc, Kristjánsson, & Skúlason, 2016), with smaller embryos often developing faster than larger embryos (Cogliati et al., 2018; Leblanc et al., 2011; Valdimarsson, Skúlason, & Snorrason, 2002). Despite the widely accepted view that egg size can affect offspring fitness (Bernardo, 1996; Mousseau & Fox, 1998), only one study has reported a link between egg size and offspring gene expression in fishes. Segers et al. (2012) found the expression levels of the growth hormone receptor (*GHR*) to be lower in smaller than larger cichlid embryos (originating from small and larger eggs, respectively), a pattern that was reversed after hatching. Such egg size‐dependent gene expression demonstrates the potential of maternal effects to influence developmental plasticity.

Finally, the model of evolution of resource polymorphism (alternative phenotypes utilising different resources; Smith & Skúlason, 1996) proposes that phenotypic plasticity is important during early stages of diversification (e.g., Schneider & Meyer, 2017; Skúlason, Snorrason, & Jonsson, 1999), and this may involve egg size‐mediated maternal effects (Benhaïm et al., 2003; Cogliati et al., 2018; Leblanc et al., 2011). Resource polymorphism is common in fishes, such as Arctic charr (*Salvelinus alpinus*), that inhabit young freshwater systems of the Northern hemisphere − likely due to lack of predation and increased intraspecific competition for habitats and food resources promoting character release (Skúlason & Smith, 1995). Although the role of developmental plasticity in diversification has been less studied than the role of genetic and ecological factors, it is becoming increasingly recognised as a key factor in the generation of polymorphism (Pfennig et al., 2010; West‐Eberhard, 2003). In this vain, Ahi et al. (2014) found gene expression networks that are associated with craniofacial divergence in developing heads of benthic and pelagic Arctic charr morphs. This gene expression variation is also reflected − to some extent − in morphology (Kapralova et al., 2015), and could explain variable timing of ossification during embryonic development in the offspring of benthic and pelagic morphs of Arctic charr (Eiríksson, Skúlason, & Snorrason, 1999).

Building upon previous findings, we aim to further understanding of patterns of gene expression during early development and how this variation relates to individual size − an important fitness trait − using a so called “brown” Arctic charr morph (BM) from lake Vatnshlíðarvatn, NW Iceland (Jónsson & Skúlason, 2000). This morph is a good candidate for studies of maternal effects and ontogenetic variation in gene expression because: 1) egg size is highly variable within and between females of similar sizes (Beck et al., in prep) and 2) it is believed to be at the early stages of divergence from another (so called “silver”) morph in this lake (Jónsson & Skúlason, 2000; Parsons, Skúlason, & Ferguson, 2010).

We tested the mRNA levels of 14 growth and/or skeletal promoting genes from post fertilization to first feeding, relative to two reference genes. We will refer to these relative differences in gene expression simply as gene expression. The growth‐related candidate genes were selected based on a literature search, whilst skeletal‐related genes were chosen to expand upon previous findings (Ahi et al., 2014; see below for further details). We studied patterns of gene expression across four developmental stages to test whether expression of growth‐related genes is higher in larger offspring (Segers et al., 2012) and whether, in contrast, expression of genes related to skeletogenesis is higher in smaller offspring (Ahi et al., 2014). These predictions are based on the hypotheses that larger individuals (i.e., originating from larger eggs) invest more energy toward growth, whilst smaller offspring (i.e., originating from smaller eggs) invest more in ossification (Eiríksson et al., 1999; Segers et al., 2012). The latter is expected because lower maternal investment in yolk should result in embryos prioritizing development of essential skeletal structures such as those related to external feeding. We assume that differences in mRNA levels at post fertilization stage − before the embryos genome has been activated (i.e., before the mid‐blastula transition, MBT; Kane & Kimmel, 1993; Nagasawa, Fernandes, Yoshizaki, Miwa, & Babiak, 2013) − will reflect maternally deposited mRNA in the egg. Due to the strong correlation between egg size and offspring size (Leblanc et al. unpublished data, see below), we will hereafter refer to “size” as encompassing both pre‐hatched and hatched offspring. We document size‐correlated changes in gene expression at the individual level and across multiple developmental stages, providing a powerful approach to determine the importance of offspring size for developmental variation in gene expression.

## 2 MATERIALS AND METHODS

### 2.1 Study system

Vatnshlíðarvatn is a physically simple, small (∼70 ha) and shallow (mean depth 2‐3 m) lake (Jónsson & Skúlason, 2000). Arctic charr is the only fish species in the lake and has diverged into two morphs (silver and brown). The brown morph has a relatively benthic shape, with a deeper body, and a more pronounced subterminal mouth (Parsons, Sheets, Skúlason, & Ferguson, 2011). In this morph, egg diameter and standard length of the embryos at hatching and first feeding are strongly correlated (at hatching: *R* ^2^ = 0.96,; at first feeding: *R* ^2^ = 0.97; *N*_(females)_ = 12 and *N*_(offspring)_ > = 168; both *P* <0.001; Leblanc et al., unpublished data).

Mature females (*N* = 7) and males (*N* = 5) were caught using gill nets (15–19 mm) in early September 2014 and 2015. All females were mated to a single male, but a subset of (*N* = 3) females shared the same male. Although this design causes variation in the relatedness of offspring, it was used to minimize potential direct genetic effects. Eggs and milt were collected and mixed in the field, where fertilized eggs were allowed to water‐harden before transport to Hólar University College's aquaculture facilities in Verið, Sauðárkrókur. Fishing in Vatnshlíðarvatn was done with permission obtained from the owner of the land (N.B. ethics’ committee approval is not needed for regular or scientific fishing in Iceland; the Icelandic law on Animal protection, Law 15/1994, last updated with Law 157/2012). Sampling was performed by Hólar University College Aquaculture Research Station (HUC‐ARC) personnel. HUC‐ARC has an operational license according to Icelandic law on aquaculture (Law 71/2008), which includes clauses of best practices for animal care and experiments.

Embryos were reared in plastic cages (6.5 cm × 6.5 cm × 5 cm) with 2 cm × 2 cm holes at the front and rear. The holes and the bottom of the cage were covered with mesh to enable good oxygenation. Cages were placed in a shelf incubator system (MariSource 8‐tray Vertical Incubator) with a constant flow of 95% recycled water. For most families, a single cage was used, but when eggs were numerous they were split into different cages to assure only one layer of eggs per cage for sufficient oxygenation. To mimic natural conditions, embryos were reared in darkness at 3.5–4.5 ºC. Temperature was manually checked on a daily basis and recorded using HOBO loggers (to the nearest 0.01 °C) four times daily. Dead individuals (opaque eggs) were manually removed every 2 days to prevent fungal growth. An accumulative temperature estimate (degree days, DD; Pruess, 1983) was used to determine the approximate time by which developmental stages would occur (see below).

### 2.2 Study design

Gene expression of Arctic charr was estimated at four developmental stages: 1) post fertilization (PF; 27DD), before the MBT (Kane & Kimmel, 1993); 2) eye stage (E; ∼200 DD), when eye lenses are formed and retinas pigmented; 3) hatching (H, ∼400 DD), when individuals have hatched but still rely on nutrition from the yolk sac; and 4) first external feeding (FF, ∼600 DD; Ballard, 1973). Sampling at each of the four stages occurred when approximately 50% of individuals within each family reached that particular stage.

At PF and E stages, individuals were visually sampled (Leblanc et al., 2011) based on their relative size (small, medium, and large) within a given clutch (Table 1). Equal numbers of individuals were taken for each relative size class and female to ensure a good representation of all egg sizes. At H and FF, individuals were chosen randomly since size selection was not feasible. A total of 5–8 individuals/family/developmental stage were collected for gene expression analyses (Table 1) and euthanised using 2‐phenoxyethanol (Pounder, Mitchell, Thomson, Pottinger, & Sneddon, 2017). All individuals (eggs, or left side of H and FF embryos) were digitally photographed (Canon EOS 650D) with a length scale, and measured for egg diameter or standard length (SL; Leblanc et al., 2016) using the program Fiji (Schindelin et al., 2012). Individual egg size was determined at PF and E stages by taking the average of four diameter measurements for each egg. All size measurements were taken to the nearest 0.001 mm. For egg size variation for additional females (not used in this study), see Table S1. Individuals from all developmental stages were then stored in an RNA stabilizing buffer (De Wit et al., 2012) and pre‐incubated at room temperature before storage (−20 ºC).

**Table 1:** Female and offspring measurements ± standard deviation (SD) of Arctic charr (*Salvelinus alpinus*) from Lake Vatnshlíðarvatn brown morph, sampled across four developmental stages: post fertilization (PF); eye stage (E); hatching (H); first feeding (FF)

Female				Offspring							
				PF		E		H		FF	
Female identity	Fork length (cm)	Weight after stripping (g)	Weight before stripping (g)	Mean egg diameter (mm)	N	Mean egg diameter (mm)	N	Mean SL (mm)	N	Mean SL (mm)	N
16	17.6	58.5	na	4.6 ± 0.07	5	4.5 ± 0.11	8	15.1 ± 0.59	7	17.9 ± 0.51	5
18	14.5	35	na	3.8 ± 0.1	6	3.7 ± 0.16	4	13.4 ± 0.36	6	16.6 ± 0.44	7
24	15.2	36.5	40	3.8 ± 0.08	6	3.6 ± 0.1	3	13.4 ± 0.32	5	16.4 ± 0.4	4
25	18.6	66	85	4.9 ± 0.07	5	4.9 ± 0.04	3	15.9 ± 0.27	6	19.1 ± 0.31	5
28	20.6	89	110	5.4 ± 0.11	5	5.5 ± 0.1	6	17 ± 0.28	6	20.5 ± 0.3	8
30	14.7	32	na	3.9 ± 0.1	6	4 ± 0.11	5	14.4 ± 0.34	6	17.7 ± 0.7	7
31	14.2	30	40	4 ± 0.08	6	4 ± 0.08	6	14 ± 0.33	4	16.2 ± 0.69	6

Sample sizes differ due to loss of samples during RNA extractions. Degree days were the same within stages (PF = 27; *E* = 242; *H* = 473; and FF = 701). na = data not available. Mean individual size (SL, standard length) and standard deviation (SD) of offspring used in this study were measured for all females. Samples sizes (N) represent all individuals for which RNA was successfully extracted.

### 2.3 RNA extraction and cDNA synthesis

Gene expression was measured using qPCR for the whole embryo at PF, E, and H stages (see Table 1 for sample sizes). Due to their large size, FF individuals were decapitated behind the pectoral fin and RNA extracted from the head and body separately. However, only the head was included in this study as much of the phenotypic variation in Arctic charr is associated with trophic morphology, as well as large body size being reflected by large head size (Kapralova et al., 2015).

Tissues were homogenized using Bead Beater (Biospec) and total RNA extracted using TRI reagent (Sigma–Aldrich, St Louis, MO). RNA extraction and cDNA synthesis were conducted according to Ahi et al. (2013). RNA was precipitated using isopropanol, washed with ethanol and air‐dried. The RNA pellet was resuspended in RNase‐free water and the RNA yields quantified using NanoDrop ND‐1000 UV/VIS spectrophotometer (NanoDrop Technologies, Wilmington, DE). The samples were treated with DNase I (New England, Biolabs, Ipswich, MA) to remove any contaminating DNA. A subset of extracted RNAs were electrophoresed on agarose gels to test RNA quality. Single stranded cDNA was synthesized from 1 µg of total RNA, using the High Capacity cDNA Reverse Transcription kit (Applied Biosystems, Foster City, CA) according to the manufacturer's protocol. cDNA was consequently diluted in nuclease‐free water in preparation for qPCR.

Fourteen genes were chosen for analyses of early life‐stage gene expression based on their previously demonstrated involvement in growth or skeletal development (see below). Two validated early developmental Arctic charr reference genes (*Actb* and *Ef1a*) were chosen for qPCR data normalisation based on Ahi et al., (2013). The following target growth‐promoting genes were selected, together with components of the glucocorticoid signaling pathway (mediating energy mobilization and stress responses): *Star* is associated with steroid and cortisol synthesis and involved in the stress response of fishes (Alsop & Vijayan, 2008); *Igf1* and *Igf2* are insulin‐like growth factors (IGFs) that play important roles in regulating growth and development in vertebrates (Greene & Chen, 1999); *Nr3c1*–here called *Gr* −, is a glucocorticoid receptor and mediator of corticosteroid signalling, highly abundant in ovulated oocytes and crucial for later skeletogenesis, particularly in the craniofacial skeleton (Pikulkaew et al., 2011); *Mtor*, mechanistic target of Rapamycin, is a key regulator of metabolism, cell growth, survival, and proliferation, and also plays an essential role in embryonic growth and development (Land, Scott, & Walker, 2014; Laplante & Sabatini, 2009); *Sgk1*, regulated by mTOR via phosphorylation and also by *Gr* through direct transcriptional activation (John et al., 2008), is a serum glucocorticoid kinase that up‐regulates ion channels in *Xenopus sp*. oocytes (Lang & Shumilina, 2013), and is involved in cell survival and postembryonic control of development in *Caenorhabditis elegans* (Jones, Greer, Pearce, & Ashrafi, 2009); *Rictor* shares a pathway with *Sgk1* and is involved in regulation of fat storage, size and development in *C. elegans* (Jones et al., 2009); *Ghr1* is a growth hormone receptor that is transcribed in very early embryonic stages and suggested to be an important developmental growth factor in vertebrates (Pierce, Breves, Moriyama, Uchida, & Grau, 2012; Sanders & Harvey, 2004). The six target genes involved in promoting skeletogenesis (*Timp2*, *Ets2*, *Sparc*, *Ctsk*, *Mmp2*, and *Mmp9,* see Table S2) were selected based on their differential expression in divergent Arctic charr morphs from Lake Thingvallavatn, Iceland (Ahi et al. 2014). These extra‐cellular matrix (ECM) remodeling genes are essential for skeletogenesis (with roles in trophic skeletogenesis) and make up a highly conserved co‐expression network across vertebrates, regulated by transcription factors such as *Ets2* (Ahi, 2016).

### 2.4 qPCR and normalization

Primers (Table S2) were designed using an assembled Arctic charr transcriptome (Guðbrandsson et al., 2018) and exon boundaries mapped to *Salmo salar* orthologs from the salmonid species database (Di Genova et al., 2011). Primers spanned at least one exon boundary and were selected based upon their short amplicon size (< 250 bp). Primer efficiencies were calculated (Table S2) and both RT‐qPCR and differential mRNA gene expression calculations for each target gene performed according to Ahi et al. (2014), using the reference genes *Actb* and *Ef1a*. These reference genes were normalized by randomly selecting one individual within each developmental stage as a calibrator sample. Relative expression quantities (RQ) were calculated according to Ahi et al. (2014).

### 2.5 Statistical analyses

All statistical analyses of gene expression were conducted in R version 3.3.2, (R Core Team, 2016) on log_2_ transformed RQ values. Normality was investigated by examining the residuals of each model using QQ plots and histograms. To control for non‐independence of data from a given family and cage, both female identity and cage were included in all models as a random intercept. Cage was not nested within female since some females had small clutches and therefore only a single cage. Finally, Pearson's correlation coefficients were calculated per developmental stage to determine the extent to which genes may be co‐expressed (see Fig. S1).

### 2.6 Developmental stage effects

We initially ran a model to test for effects of developmental stage, gene identity and their interaction (individual size was not included in this model as size is confounded with developmental stage) on log_(2)_ transformed relative mRNA expression values (note, however, that any differences in gene expression at FF compared to other developmental stages may in part reflect sampling procedures as RNA was extracted from the whole embryo at PF, E, and H, but only from the head at FF stage). Due to significant interactions between developmental stage and gene identity (see Table S3), subsequent analyses were conducted within each gene separately using linear mixed effects models (LME) by fitting the *lmer* function from the *lme4* package (Bates, Mächler, Bolker, & Walker, 2015). In these models, developmental stage was used as a fixed effect, female and cage as random effects. Post‐hoc Tukey adjusted population Least square means (LS means) and pairwise comparisons were calculated for developmental stage effects using the functions *lsmeansLT* and *difflsmeans*, respectively, from the *lsmeans* package (Lenth, 2016).

### 2.7 Size correlated gene expression

To test for dependency of gene expression on individual size, a LME model was fitted per developmental stage. These models included gene identity (14 levels) as a fixed factor (as above) and individual size, as well as its interaction with gene identity, as a covariate. In all models, female identity and cage were included as random effects in the full model. The *anova* function was used to determine whether the best fitting model (within a given developmental stage) was linear or polynomial (second or third order) using Akaike Information Criterion (AIC). Subsequent analyses were then conducted for the best fitting model, whereby (for both linear and non‐linear models) if the size × gene identity interaction was significant, the slope of gene expression and individual size was compared across all genes to determine how expression of genes was correlated with size using the *emtrends* “*pairs*” function from the estimated marginal means (*emmeans*) package (Lenth, 2017). The *test* function was then used to determine which slopes were significantly different from zero.

## 3 RESULTS

Average egg size per female (*N* = 7) was 4.3 ± 0.09 mm at PF, 4.3 ± 0.1 mm at E, and average standard length of embryos was 14.7 ± 0.36 mm at H and 17.8 ± 0.48 mm at FF (Table 1).

### 3.1 Developmental stage effects on gene expression

**Figure 1:**
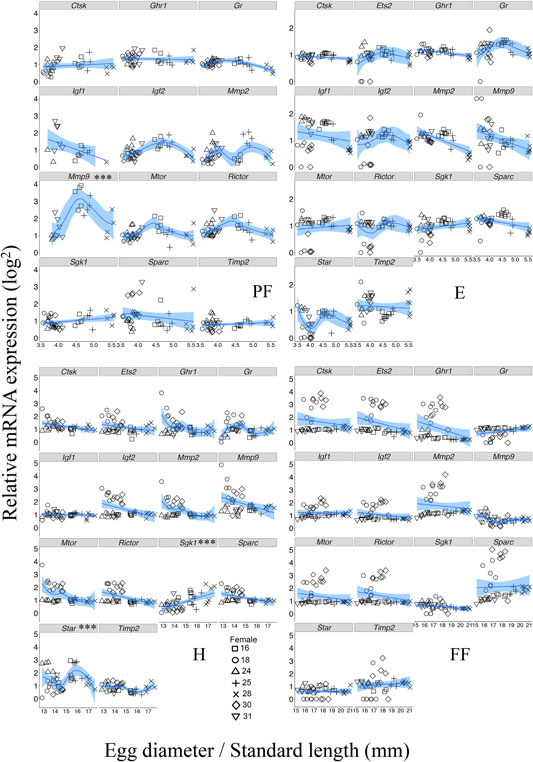
Relative gene expression correlations with egg/individual size at post fertilization (PF), eye stage (E), hatching (H) and first feeding (FF) in Arctic charr (*Salvelinus alpinus*) from lake Vatnshlíðarvatn. Note that at PF, two genes (*Star* and *Ets2*) were not detected by qPCR and have been removed from the figure. Both linear and non‐linear fits were tested initially for each gene (see Methods section) and the results of the best fitting model represented here. Genes that are significantly correlated with size are *Mmp9* at PF, and *Sgk1* and *Star* at H (which best fitted non‐linear, linear and non‐linear models, respectively, see Table S4). Significance levels are indicated in the gene names of the plots by: *p* *< 0.01, ** < 0.001 and *** < 0.0001. Symbols correspond to different female identities (i.e., different clutches)

**Figure 2:**
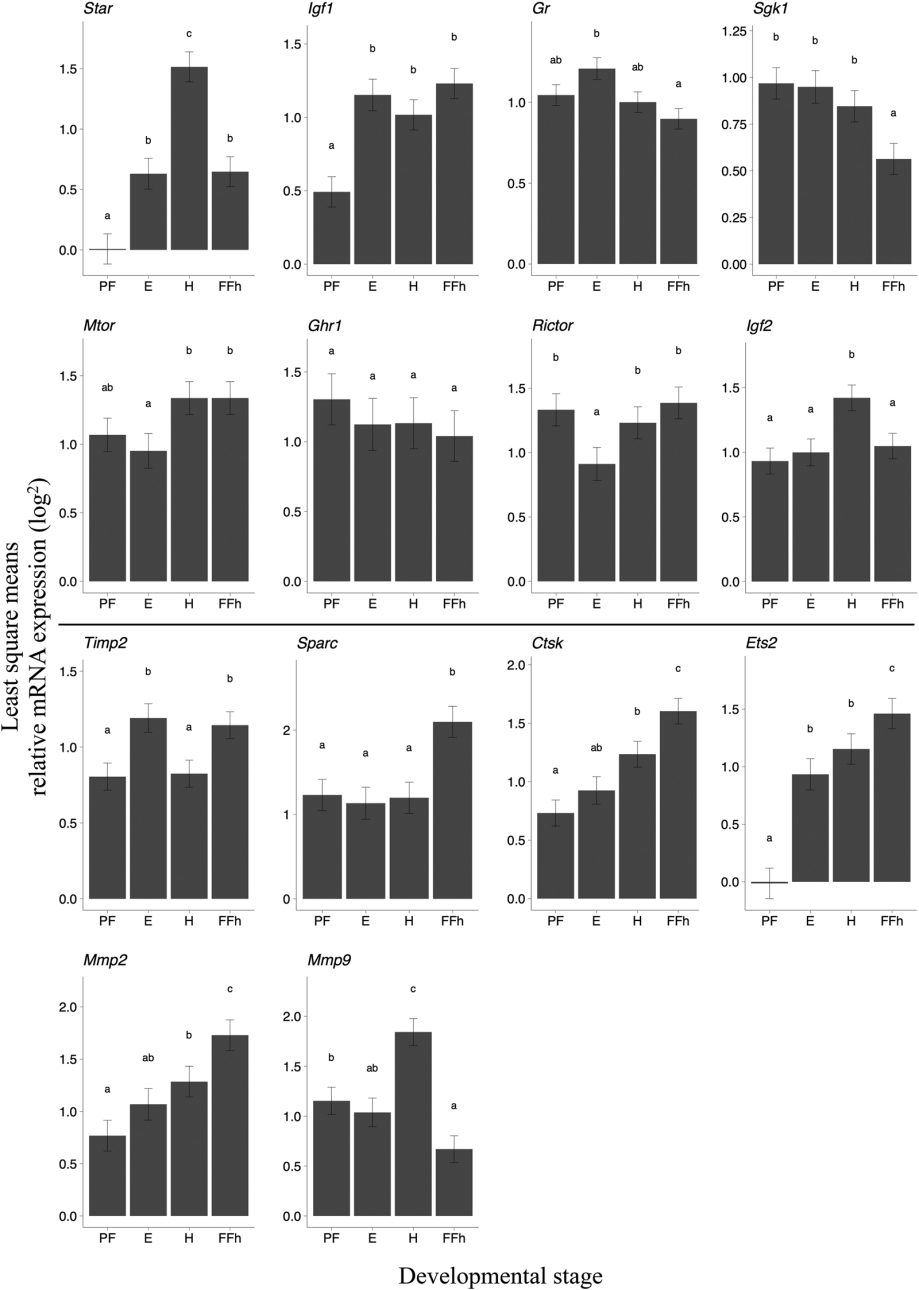
Least square (LS) means ± S.E. of relative mRNA expression at four developmental stages: post fertilization (PF), eye stage (E), hatching (H) and first feeding (FF) in Arctic charr (*Salvelinus alpinus*) from lake Vatnshlíðarvatn. All LS means are significantly different from zero, with the exception of *Star* and *Ets2* at PF stage (not expressed). Least‐square means sharing a letter are not significantly different from each other (Tukey‐adjusted pairwise comparisons of LS means). Above the centerline are growth related genes, and below are genes involved in skeletogenesis

There was a significant gene × developmental stage interaction on relative expression levels (*F* = 14.03; Num DF = 39; Den DF = 2094.4; *p* <0.0001; Table S3). Least‐square means of gene expression differed from zero for all genes and developmental stages (genes: *p* <0.0001), with the exception of *Ets2* and *Star*, which were not detected at PF (Figure 1). *Igf1* expression was detected only in a few individuals at PF (*n* = 12; 31%), almost half of which were offspring of the same female (ID = 31). Gene specific LME analyses (Table 2) revealed that gene expression levels varied strongly among developmental stages for almost all genes (all *p* < 0.004), apart from *Ghr1*, which was consistently highly expressed (developmental stage: *p = *0.172; Table 2 and Figure 2).

**Table 2:** Linear mixed effects model used to test the effect of developmental stage on gene expression (log expression) in Arctic charr from lake Vatnshlíðarvatn

		Female (Random Effect)		Developmental stage (Fixed Effect)		
Gene identity (growth)	*N*	*X* ^2^	*P*	Sum Sq	*F*	*P*
*Star*	155	11.10	**0.001**	45.30	90.52	<0.0001
*Igf1*	155	8.02	**0.005**	12.83	19.92	<0.0001
*Gr*	155	1.16	0.300	1.86	4.57	0.004
*Sgk1*	155	5.42	**0.020**	4.14	9.64	<0.0001
*Mtor*	154	7.94	**0.005**	4.21	4.74	0.004
*Ghr1*	154	13.80	**0.000**	1.44	1.69	0.172
*Rictor*	154	10.20	**0.001**	4.88	5.97	<0.001
*Igf2*	155	5.61	**0.020**	5.67	8.00	≤0.0001
Gene identity (skeletal)						
*Timp2*	154	4.32	**0.040**	4.72	8.90	<0.0001
*Sparc*	155	11.30	**0.001**	24.87	18.66	<0.0001
*Ctsk*	154	3.81	**0.050**	17.11	18.13	<0.0001
*Ets2*	155	10.70	**0.001**	47.78	62.98	<0.0001
*Mmp2*	154	11.70	**0.001**	19.45	19.81	<0.0001
*Mmp9*	155	0.28	0.600	28.94	14.36	<0.0001

N: number of individuals across all developmental stages. *χ* ^2^: Chi square statistic. *P*: *P*‐value (for random effects reflect the likelihood ratio test). Dev.stage: developmental stage. Sum Sq: sum of squares. Den DF: denominator degrees of freedom based on Satterthwaite's approximations. F: *F*‐value. Significance of random (female, *n* = 7) and fixed (developmental stage) effects included for the LME model. Significant results (*P* < 0.05) indicated in bold. Cage was non significant in all cases. Degrees of freedom (DF) of Chi square test and numerator DF were 1 and 3, respectively, in all cases.

Least‐square mean expression of three ECM remodeling genes important for skeletogenesis (*Ctsk*, *Ets2*, and *Mmp2*) tended to increase throughout ontogeny, although expression at E stage was not different from PF and H stages (Tukey pairwise *p* > 0.05 for both; Figure 2). For *Sgk1*, relative expression seemed to decrease throughout ontogeny (Figure 2), however, only the drop at FF was significant. The random effect of family was significant in most cases (Table 2), indicating family level variation in gene expression.

### 3.2 Individual size correlated gene expression

Polynomial models of egg size correlated gene expression showed a better fit than a linear model at all developmental stages (see Table S4 for model comparisons and Table 3 for results). Female identity generated much of the variation in gene expression at PF (Figure 1) in a linear model, but this effect was non‐significant under a polynomial model (see Table 3). The size × gene identity interaction was significant at all developmental stages (Table 3). This arose in part because relative expression of *Mmp9* (at PF), *Sgk1*, and *Star* (at H) was correlated with size (all *p < *0.0001, Table 3). However, when analyzing the effect of size on individual genes using the best‐fitting (linear or non‐linear) model for each gene separately, *Sgk1* had significantly higher expression in larger embryos at H stage (linear model; see Table S4), whereas individual size was non‐linearly related to expression of *Mmp9* and *Star* (Figure 1). *Post‐hoc* pairwise comparisons of gene expression (data not shown) revealed that: 1) at PF, *Mmp9* had higher expression than all other genes (*p *<0.0001); 2) at E stage, *Timp2* showed lower expression in comparison to *Mtor* (*t *= −3.82, *p *< 0.05) and *Star* (*t *= −4.08, *p* < 0.01); 3) at H, *Star* also had higher expression than all other genes (*p *< 0.05) except *Sgk1,* which showed significantly higher expression (fitting a non‐linear model) than both *Rictor* and *Gr* (both *t *= 0.62, *p* < 0.05); and finally, 4) no differences in expression levels were found at FF. When examining gene co‐expression across all developmental stages (Figure S1), we find very few weakly co‐expressed at PF, which increases at E stage where more growth‐related genes are co‐expressed than genes involved in skeletogenesis. At H stage, growth related genes such as *Star* and *Sgk1* have a weak positive correlation − but are negatively correlated with all other genes (similar to findings from Ahi et al., 2014), whilst most genes associated with skeletogenesis showed very strong co‐expression. This pattern somewhat remains at FF, where the main differences is the drop in co‐expression of *Mmp9*. Throughout all developmental stages, *Timp2* does not show any strong correlations with any of the genes used in this study (Figure S1).

**Table 3:** Results of non‐linear mixed effect models testing for the effect of gene identity (fixed factor) and individual size (covariate), as well as their interaction, on gene expression (see Table S5 for results using linear mixed effects models) in Arctic charr from lake Vatnshlíðarvatn

Dev. stage	*N*	Response	Variable	Variance	SD	*X* ^2^	*P*	Variable	Sum Sq	Num DF	Den DF	*F*	*P*	*β*	SE	DF	*t*	*P*
**PF**	546	Log Expression	Female	0.014	0.12	2.66	0.103	Size (poly2)	1.85	2	6	4.56	0.064					
			Cage	0.001	0.04	0.35	0.556	**Genes**	94.22	13	489	35.71	**<0.0001**					
								Size*Genes	38.31	26	489	7.26	**<0.0001**					
								***Mmp9***						2.16	0.191	114	11.31	**<0.0001**
**E**	485		**Female**	0.05	0.22	5.97	**0.015**	**Size (poly3)**	1.4	3	14	5.23	0.013					
			**Cage**	0.008	0.09	4.13	**0.042**	**Genes**	10.71	13	410	9.25	**<0.0001**					
								Size*Genes	11.16	39	410	3.21	**<0.0001**					
**H**	559		**Female**	0.07	0.26	8.13	**0.004**	Size (poly3)	0.22	3	85	0.37	0.778					
			**Cage**	0.009	0.09	6.58	**0.01**	**Genes**	37.73	13	488	14.27	**<0.0001**					
								Size*Genes	29.76	39	488	3.75	**<0.0001**					
								***Star***						0.69	0.137	432	5.02	**<0.0001**
**FF**	574		**Female**	0.185	0.43	15.09	**<0.001**	Size (poly2)	0.73	2	76	1.06	0.353					
			Cage	0.006	0.08	1.54	0.215	**Genes**	106.78	13	520	23.81	**<0.0001**					
								Size*Genes	27.3	26	520	3.04	**<0.0001**					

Dev.stage; developmental stage. N; total number of observations across all individuals and genes. Log expression; log2 (relative mRNA expression + 1). *X* ^2^; Chi square statistic. Chi. DF; number of degrees of freedom for the test. *P*; *P*‐value of the likelihood ratio test for the random effect. Sum Sq; sum of squares. Num DF; numerator degrees of freedom. Den DF; denominator degrees of freedom based on Satterthwaite's approximations. F; F‐value. Gene slopes that remained significantly different from zero after Tukey's adjustments are indicated, as well as slope (β), standard error (SE), degrees of freedom (DF), t‐ratio (*t*) and associated *P*‐values. Significant variables in bold. PF; post fertlization. E; eye stage. H; hatching stage. FF; first feeding stage. These analyses were conducted separately for each developmental stage due to significant differences among developmental stages (see Table S3). Significance of random (female and cage) and fixed effects are included for the overall models.

## 4 DISCUSSION

Out of the 14 genes related to growth and/or skeletal development that we analyzed, transcripts of 12 genes were present already at post fertilization (i.e., before the mid‐blastula transition, MBT). Expression of only three of the genes correlated with offspring size: *Mmp9* at post fertilization, and *Sgk1* and *Star* at hatching stage. Moreover, *Sgk1* (a growth‐related gene) showed higher expression in larger offspring, whilst expression of *Mmp9* and *Star* was non‐linear in relation to offspring size. These findings are somewhat counter to our predictions (with the exception of *Sgk1*; see below) but do suggest that egg size‐correlated expression of genes crucial for skeletogenesis and growth may influence plasticity during early development.

### 4.1 Evidence for trophic development

The diversification of trophic structures in teleost fishes has not only resulted in morphologies that match feeding niches (Skúlason & Smith, 1995), but have also facilitated adaptive radiations (Muschick, Indermaur, & Salzburger, 2012). Numerous molecular processes are involved in the development of the vertebrate feeding apparatus (Ahi, 2016), some of which have been involved in the diversification of sympatric morphs of Arctic charr (Ahi et al., 2014). For example, *Mmp9*, *Mmp2*, *Ctsk*, *Timp2*, and *Sparc* are all part of the same conserved co‐expression network involved in bone remodeling, with *Ets2* identified as a potential regulator (Ahi et al., 2014). These genes also seem to be showing similar expression patterns in our study at hatching stage, particularly the strong negative correlated expression of both *Star* and *Sgk1* with all other genes (Figure S1c). At hatching, we also find a large increase in the expression of *Mmp9*. The glucocorticoid pathway is involved in the regulation of both *Mmp9* and *Mmp2* during craniofacial skeletogenesis, altering morphogenesis of the pharyngeal cartilages (Hillegass, Villano, Cooper, & White, 2008). *Mmp9* transcripts have been reported in pharynx cells in zebrafish (*Danio rerio*) embryos from 2‐5dpf (Sharif, de Bakker, & Richardson, 2014) and osteogenesis (i.e., transformation of cartilaginous skeleton transforms into calcified tissue) is known to occur by hatching (Du, Frenkel, Kindschi, & Zohar, 2001). The involvement of this gene in the development of skeletal elements in the pharyngeal jaw of zebrafish also suggests its likely role in trophic development in Arctic charr (Ahi et al., 2014).

By the critical first feeding stage, we find the gradual increase in expression of *Ctsk* in the head to be at the highest. *Ctsk* has a similar role to *Mmp9* in osteogenesis and is important for both calcification (Du et al., 2001) and bone remodeling (Petrey et al., 2012). Thus strongly suggesting the potential for this gene to facilitate plasticity of the trophic apparatus in response to diet (e.g., Parsons et al., 2011), especially during first feeding (Figure 2). A gradual increase in expression throughout development was also reflected by *Mmp2* (Figure 2), indicating an increased need for ECM remodeling and tissue morphogenesis, such as needed for muscle growth and swimming physiology (Michelin et al., 2009), all of which are crucial for survival after hatching. Three genes involved in bone remodeling (*Ets2*, *Sparc*, and *Timp2*) also increased at the onset of active feeding (Lima, Andrade, Pini, Makrakis, & Makrakis, 2017). It is worth noting the higher expression of genes involved in skeletogenesis may reflect previous findings suggesting that benthivoros charr embryos tend to allocate more energy toward bone development than do pelagic planktivorous charr embryos (Eiríksson et al., 1999). Although direct comparisons of gene expression between the two sympatric morphs found in lake Vatnshlíðarvatn are needed, our current results do demonstrate that the more benthic “brown” Arctic charr morph has − overall − relatively high expression levels of genes related to skeletogenesis throughout ontogeny in comparison to those genes related to growth. The role of these genes in the early development of morphological variation, their sensitivity to different diets (Parsons et al., 2011) and association with egg and embryo size, clearly warrants further attention.

### 4.2 Other relevant patterns of gene expression

The transition of embryos from the egg to the external environment requires important physiological changes. One such change that occurs is that of increased larval cortisol, which is essential for an individuals’ ability to cope with stress (Mommsen, Vijayan, & Moon, 1999). Our study here, as well as studies on zebrafish, show mRNA levels of *Star* to be up‐regulated immediately before a dramatic rise in basal larval cortisol after hatching (Alsop & Vijayan, 2009). Such elevation may indicate stress induced by hatching. Indeed, evidence from both Rainbow trout (*Oncorhynchus mykiss*) and zebrafish (Barry, Malison, Held, & Parrish, 1995) suggest that de novo synthesis of this steroid occurs around hatching and, based on our results, this also seems to be the case in Arctic charr.

### 4.3 Maternal influence on gene expression at post fertilization

Maternal influence on embryonic development persists until the switch from maternal to embryonic control of gene expression, which occurs at the MBT at 7 days post fertilization (henceforth dpf; 42 DD) in Atlantic salmon (*Salmo salar*; Nagasawa et al., 2013). Our post fertilization samples were collected 4 dpf (27 DD) and hence prior to the MBT in salmonids. Nevertheless, there is evidence from other studies that zygotic transcripts of genes that are not maternally expressed, can be detected prior to the expected activation of the embryonic genome, that is, for the clearance of maternal mRNAs (Lund, Liu, Hartley, Sheets, & Dahlberg, 2009; Yang, 2002; Zhang, 2003). In the literature on other taxa, we find evidence for pre‐MBT transcriptional activity of all genes used in this study, with the exception of *Sparc*. In our study, the expression of *Sparc* occurred before the MBT, however, studies on *Xenopus* do not detect this gene until late gastrulation (Damjanovski, Huynh, Motamed, Sage, & Ringuette, 1998), we therefore suggest that expression of *Sparc* at post fertilization, may be a product of zygotic transcription. Out of the 14 genes analysed in our study, transcripts of *Ets2* and *Star* were not detectable at the post fertilization stage, strongly suggesting that transcripts of these two genes are not deposited in the egg, but are instead produced by the embryonic genome later in development. In accordance, *Star* was not expressed until the eye stage in zebrafish (Alsop & Vijayan, 2008). Furthermore, down‐regulation of *Star* occurs in response to excess cortisol (Nesan & Vijayan, 2016), the primary circulating glucocorticoid in fish, whereby the glucocorticoid receptor (*Gr*) can modulate transcriptional activity of transcription factors, such as *Ets2* (Ahi, 2016; Hill, Sussan, Reeves, & Richtsmeier, 2009), which was also found to be undetectable in our study at post fertilization.

### 4.4 Evidence for the role of maternal effects

We provide some evidence for egg/offspring size being correlated with gene expression (*Mmp9*, *Star*, and *Sgk1*), but two out of three genes (*Mmp9* and *Star*) contrasted with our original prediction of larger offspring expressing more growth related genes, whilst smaller offspring more skeletal development genes. Previous studies found *Sgk1* expression levels to be lower during craniofacial skeletal development in benthic compared to pelagic Arctic charr (Ahi et al., 2014). Here we find lower expression of *Sgk1* in smaller offspring (i.e., originating from smaller eggs), which − considering the differential expression of this gene in benthic/pelagic Arctic charr morphs (Ahi et al., 2014) − may be especially relevant in the context of this morph being at the very early stages of divergence. Such changes in gene expression are reflected by changes in the phenotype in several taxa (e.g., Abzhanov et al., 2006; Chan et al., 2010; Uebbing et al., 2016). For instance, previous studies found differential gene expression between sympatric pairs of fish that are often associated with differences along a benthic/pelagic axis (Ahi et al., 2014; Fudickar et al., 2016; Guðbrandsson et al., 2018). Furthermore, differences in ossification and growth rates have been found in sympatric Dolly Varden (*Salvelinus malma*: Esin, Markevich, & Pichugin, 2018), concomitant to variation in expression of genes involved in the regulation of growth and skeletogenesis (our study). Thus highlighting the need for targeted studies that explicitly link development and growth with underlying patterns of gene expression. Although we find evidence for the role of maternal effects (egg size) in influencing the expression of three genes involved in growth and skeletogenesis, further evidence is clearly needed to make general predictions regarding egg size correlated gene expression.

## 5 CONCLUSION

Our results provide evidence for dynamic variation in expression of important genes related to growth and skeletogenesis across early life‐stages. We also find some evidence for this variation being correlated with offspring size (for *Mmp9* at post fertilization, and *Sgk1* and *Star* at hatching). Interestingly, all three of these genes are differentially expressed in benthic and pelagic Arctic charr morphs (Ahi et al., 2014), suggesting potential for diversification of different phenotypes during early life‐stages.

Better understanding the causes of phenotypic divergence at early life‐stages and the relationship between egg size and offspring phenotype and fitness − especially under differing dietary/ecological conditions − would shed light both on early stages of divergence along the speciation continuum (e.g., Nosil, 2012) as well as the evolution of optimal maternal investment in contrasting environments (e.g., Smith & Fretwell, 1974). The number of studies documenting egg size mediated effects on offspring phenotype in fishes is rapidly growing (Cogliati et al., 2018; Leblanc et al., 2011; Leblanc et al., 2016; Segers et al., 2012; Self, Schreck, Cogliati, Billman, & Noakes, 2018; Thorn & Morbey, 2018). At the same time, our knowledge as to whether egg size can influence evolutionary divergence is limited. Future studies should address this knowledge gap by combining data on offspring gene expression and phenotypes (e.g., morphology and behavior) across developmental stages in multiple populations along the diversification continuum (i.e., incorporating populations both at early stages of divergence and well‐diverged populations). For instance, egg size variation along such a continuum may decline with increasing diversification due to canalization (Parsons et al., 2011; Waddington, 1942), and strong divergence in egg size is also likely in populations inhabiting contrasting environments. Environmental heterogeneity can influence egg size (Koops, Hutchings, & Adams, 2003), and could therefore be considered a factor in the generation of different phenotypes especially given the potential for egg size mediated differences in gene expression (Segers et al., 2012), as well as phenotype (e.g., Cogliati et al., 2018). In general, our findings on gene expression suggest that maternal effects and early developmental changes in gene expression may influence developmental plasticity, yet studies combining both gene expression and phenotypic variation are needed to further our understanding on the mechanisms of early phenotypic divergence.

## Supporting information

Additional supporting information may be found online in the Supporting Information section at the end of the article.

Supporting Information S1.
