# Supplementary material for: Gene expression in the phenotypically plastic Arctic charr (Salvelinus alpinus): A focus on growth and ossification at early stages of development
Source: Evol Dev. 2018 Nov 26;21(1):16–30. doi: 10.1111/ede.12275 (PMC9285049; doi:10.1111/ede.12275)
Supplement: Supplementary file 1 — Supporting Information S1. [file EDE-21-16-s001.docx]

**Figure S1** Pearsons’ correlation coefficients between expression levels of 14 genes related to growth (*Star/Igf1 – Igf2*) and skeletogenesis (*Timp2* – *Mmp9*), across four developmental stages in the brown morph of Arctic charr from lake Vatnshlíðarvatn: a) post-fertilisation (PF), b) eye stage (E), c) hatching (H), and d) first feeding (FF). Note that at PF, two genes were not detected by qPCR (*Star* and *Ets2*) and panels at this developmental stage are therefore in a different order in comparison to E, H and FF.

Numbers indicate Pearson’s pairwise correlation coefficients between expression levels of seven females of Arctic charr at each developmental stage. Blue represents positive and red represents negative expression correlation. A scale showing critical values of *r* is depicted with corresponding colours. Critical values of *r* for PF is different to the other developmental stages as two genes (*Star* and *Ets2*) were not detected by qPCR. DF, degrees of freedom. P: *< 0.01, **<0.001 and ***<0.0001.

**Table S1** Additional female characteristics that were measured for fork length and offspring size, but not used for gene expression analysis, are shown across four developmental stages (1) post-fertilisation, PF; 2) eye stage, E; 3) hatching, H; and 4) first feeding, FF) in the brown morph of Arctic charr from lake Vatnshlíðarvatn.

| **Female** | | | | | **Offspring stage** | | | | | | | | | | | | | | | |
| --- | --- | --- | --- | --- | --- | --- | --- | --- | --- | --- | --- | --- | --- | --- | --- | --- | --- | --- | --- | --- |
|  |  |  |  |  | **PF** | | | | **E** | | | | **H** | | | | **FF** | | | |
| **Female identity** | **Year** | **Fork length (cm)** | **Weight after (g)** | **Weight before (g)** | **Mean egg diameter (mm)** | **SD** | **DD** | **N** | **Mean egg diameter (mm)** | **SD** | **DD** | **N** | **SL (mm)** | **SD** | **DD** | **N** | **SL (mm)** | **SD** | **DD** | **N** |
| 17 | 2014 | 17 | 46 |  | 5.1 | 0.08 | 27 | 6 | 5.1 | 0.17 | 242 | 6 | 16.5 | 0.07 | 473 | 6 | 20.2 | 0.87 | 701 | 10 |
| 23 | 2014 |  | 45 |  | 3.6 | 0.1 | 27 | 6 | 3.6 | 0.11 | 242 | 6 | 13 | 0.49 | 462 | 6 | 15.1 | 0.28 | 701 | 10 |
| 26 | 2014 | 13.7 | 27.5 | 30 | 3.6 | 0.05 | 27 | 6 | 3.7 | 0.07 | 242 | 6 | 12.7 | 0.59 | 473 | 6 | 14.9 | 1 | 701 | 8 |
| 135 | 2015 | 17.6 | 60 | 74 | 4.4 | 0.15 | 12 | 7 | 4.5 | 0.12 | 217 | 12 | 15.2 | 0.43 | 470 | 9 | 18.4 | 0.61 | 639 | 13 |
| 140 | 2015 | 18 | 65 |  | 4.4 | 0.12 | 12 | 8 | 4.4 | 0.08 | 230 | 5 | 15 | 0.37 | 442 | 7 | 18.5 | 0.36 | 639 | 15 |
| 143 | 2015 | 24.8 | 131 | 146 | 4.4 | 0.13 | 12 | 16 | 4.4 | 0.1 | 217 | 14 | 13.6 | 0.24 | 386 | 14 | 17.5 | 2.63 | 639 | 12 |
| 149 | 2015 | 17.2 | 54 |  | 4.4 | 0.08 | 12 | 7 | 4.4 | 0.13 | 230 | 6 | 14.3 | 0.33 | 440 | 15 | 17.5 | 0.44 | 639 | 5 |
| 150 | 2015 | 24.1 | 128 | 160 | 5.4 | 0.12 | 12 | 10 | 5.3 | 0.15 | 217 | 14 | 14.5 | 1.55 | 419 | 17 | 19.9 | 0.49 | 639 | 12 |
| 152 | 2015 | 15.7 | 44 |  | 4.2 | 0.1 | 12 | 8 | 4.4 | 0.09 | 217 | 6 | 14.3 | 0.45 | 442 | 7 | 16.5 | 0.6 | 639 | 9 |
| 154 | 2015 | 19.8 | 76 | 84 | 4.2 | 0.09 | 12 | 8 | 4.3 | 0.08 | 217 | 5 | 14.5 | 0.34 | 438 | 6 | 17.3 | 0.49 | 610 | 8 |

Mean individual size (egg diameter, mm) or (SL, standard length) of offspring (N) with associated standard deviation (SD) were measured for all females. Sample sizes (N) represent all individuals that have been preserved in an RNA stabilising buffer and measured. Blank spaces indicate missing data. Female weight before and after stripping eggs is given when available.

**Table S2** Primer sequences and relevant information for Arctic charr (*Salvelinus alpinus*)

| Gene | Description | | Primer Sequence (5’- 3’) | Product Size (bp) | PCR Efficiency | Exon boundary | Accession no. |
| --- | --- | --- | --- | --- | --- | --- | --- |
| ***Actb*** | | Beta Cytoskeletal Actin | F-GAAGATCAAGATCATCGCCC | 122 | 1.95 | Yes | JR540730 |
|  |  |  | R-CAGACTCGTCGTACTCCTGCT |  |  |  |  |
| ***Ctsk*** | | Cathepsin K | F-CAGATGAGGCTTACCCTTACCTTGG | 74 | 1.98 | Yes | LM643986 |
|  |  |  | R-ACACTGAGCACCCATGCCAG |  |  |  |  |
| ***Ets2*** | | V-Ets Erythroblastosis Virus E26 Oncogene Homolog 2 (Avian) | F-AAGACTACCCTGCTGACC | 155 | 1.92 | Yes | LM644000 |
|  |  |  | R-CTCTCCAATGAACACAAACTCTG |  |  |  |  |
| ***Mmp2*** | | Matrix Metallopeptidase 2 | F-ATGGATGGAGAGGCTGACATC | 110 | 1.94 | Yes | KC538875.1 |
|  |  |  | R-GGTCCAGGAGCAAAGGCAT |  |  |  |  |
| ***Mmp9*** | | Matrix Metallopeptidase 9 | F-AATCTACTTCTTCTCAGGCACC | 111 | 1.9 | Yes | LM643995 |
|  |  |  | R-CTCCCTCCACCTTCTCTAC |  |  |  |  |
| ***Gr*** | | Glucocorticoid Receptor | F-CGCTCCTACCAGCAGTGTA | 80 | 1.96 | Yes | LM643989 |
|  |  |  | R-GGCAACTTCATCCTCTCATCAT |  |  |  |  |
| ***Sgk1*** | | Serum/Glucocorticoid Regulated Kinase 1 | F-CCTCCGTTCTACAGTCGTAA | 113 | 1.88 | Yes | LM644002 |
|  |  |  | R-AGCAGTCCCTCCAGTATGT |  |  |  |  |
| ***Sparc*** | | Secreted Protein, Acidic, Cysteine-Rich (Osteonectin) | F-GTTCTGGTCACCCTGTACGAG | 100 | 1.92 | No | KC538874.1 |
|  |  |  | R-GCAGTCTCTTCTCATTCTCATAGATC |  |  |  |  |
| ***Star*** | | Steroidogenic Acute Regulator | F-TCTGCTTGTGTAATCATAGCCC | 104 | 1.92 | Yes | AF232215.1 |
|  |  |  | R-GTGGGAATGTGTGTATGTTTTGAG |  |  |  |  |
| ***Timp2*** | | Tissue Inhibitor of Metalloproteinase 2 | F-GTCATACTTGATCTCGTTAGACAAA | 84 | 1.94 | Yes | LM644001 |
|  |  |  | R-CAACAGGCTTTTTGCGATG |  |  |  |  |
| ***Igf2*** | | Insulin Like Growth Factor 2 | F-GGAGACGCTATGTGGAGGAGAACT | 98 | 1.9 | Yes | GU933430.1 |
|  |  |  | R-TGCTGTTAGACCTGCTGGTTGG |  |  |  |  |
| ***Ghr1*** | | Growth Hormone Receptor 1 | F-ACATCCCCACTAAAGAGTCCCGA | 123 | 1.98 | Yes | XM_014133894.1 |
|  |  |  | R-GGAGAATCACCATCAACTTCTGTTGC |  |  |  |  |
| ***Mtor*** | | Mechanistic Target Of Rapamycin Kinase | F-GGCACAGGTTCTCGTGCGA | 105 | 1.89 | Yes | XM_024135341.1 |
|  |  |  | R-GAGACAAGCTGACTGGGCGTGA |  |  |  |  |
| ***Rictor*** | | Rapamycin-Insensitive Companion Of MTOR | F-GATCCACCCGTCTCTGTCTCATAGT | 136 | 1.93 | Yes | XM_024001547.1 |
|  |  |  | R-GCTCAACGCCTTCAGAAACCTG |  |  |  |  |

**Table S3** A mixed effects model to test for the effects of developmental stage (dev. stage), gene identity (gene) and their interaction on gene expression in Arctic charr from lake Vatnshlíðarvatn. The model included female identity (N= 7) and cage as random effects to account for non-independence of data.

|  | **SS** | **MS** | **NumDF** | **DenDF** | **F** | ***P*** |
| --- | --- | --- | --- | --- | --- | --- |
| **Dev.stage** | 51.38 | 17.1 | 3 | 1900 | 53.33 | **<0.0001** |
| **Gene** | 70.74 | 5.4 | 13 | 2094 | 16.94 | **<0.0001** |
| **Dev.stage x gene** | 175.72 | 4.5 | 39 | 2094 | 14.03 | **<0.0001** |

Sum Sq; sum of squares. Num DF; numerator degrees of freedom. Den DF; denominator degrees of freedom based on Satterthwaite’s approximations. F = F-value. *P* =*P*-value to determine the significance of these fixed effects on relative gene expression (log^2^).

**Table S4** Model selection results using the Akaike Information Criterion (AIC) to determine whether gene identity (fixed factor), individual size (covariate), as well as their interaction on gene expression was best fitted using a linear or non-linear (polynomial) model across four developmental stages of Arctic charr from lake Vatnshlíðarvatn: PF, post-fertilisation; E, eye stage; H, hatching; and FF, first feeding. The model included female identity (N = 7) and cage as random effects to account for non-independence of data.

| **Dev. stage** | **Model** | **Df** | **AIC** | ***X*^2^** | ***X* Df** | ***P*** |
| --- | --- | --- | --- | --- | --- | --- |
| **PF** | Linear | 31 | 824 |  |  |  |
|  | **Polynomial^2^** | 45 | 740 | 111.91 | 14 | **<0.0001** |
|  | Polynomial^3^ | 59 | 743 | 24.36 | 14 | 0.041 |
| **E** | Linear | 31 | 325 |  |  |  |
|  | Polynomial^2^ | 45 | 304 | 49.40 | 14 | <0.0001 |
|  | **Polynomial^3^** | **59** | **297** | **34.80** | **14** | **0.002** |
| **H** | Linear | 31 | 793 |  |  |  |
|  | Polynomial^2^ | 45 | 803 | 17.71 | 14 | 0.2206 |
|  | **Polynomial^3^** | 59 | 787 | 44.12 | 14 | **<0.0001** |
| **FF** | Linear | 31 | 1116 |  |  |  |
|  | **Polynomial^2^** | 45 | 1095 | 48.89 | 14 | **<0.0001** |
|  | Polynomial^3^ | 59 | 1096 | 27.01 | 14 | 0.019 |

Dev.stage, developmental stage. Df, degrees of freedom. AIC, Akaike Information Criterion. *X*^2^, chi square.

**Table S5** Results of linear mixed effect models testing for the effect of gene identity (fixed factor) and individual size (covariate), as well as their interaction, on gene expression in Arctic charr from lake Vatnshlíðarvatn. These analyses were conducted separately for each developmental stage due to significant differences among developmental stages (see Table S3). Significance of random (female and cage) and fixed effects are included for the overall models.

| **Dev. stage** | **N** | **Response** | **Random Effects** | | | | **Fixed Effects** | | | | | | | | | | |
| --- | --- | --- | --- | --- | --- | --- | --- | --- | --- | --- | --- | --- | --- | --- | --- | --- | --- |
|  |  |  | **Variable** | **Chi. sq** | **Chi. DF** | ***P*** | **Variable** | **Sum Sq** | **Num DF** | **Den DF** | **F** | **P** | **β** | **SE** | **DF** | **t** | ***P*** |
| PF | 546 | Log Expression | **Female** | 12.733 | 1 | **<.0001** | size | 0.12 | 1 | 8 | 0.50 | 0.497 |  |  |  |  |  |
|  |  |  | Cage | 0.048 | 1 | 0.827 | **gene** | 16.54 | 13 | 505 | 5.29 | **<.0001** |  |  |  |  |  |
|  |  |  |  |  |  |  | **size*gene** | 16.53 | 13 | 505 | 5.29 | **<.0001** |  |  |  |  |  |
|  |  |  |  |  |  |  | ***Mmp9*** |  |  |  |  |  | 1.026 | 0.174 | 35 | 5.882 | **<0.0001** |
| E | 485 |  | **Female** | 14.600 | 1 | **<.0001** | size | 0.08 | 1 | 10 | 0.75 | 0.407 |  |  |  |  |  |
|  |  |  | Cage | 0.000 | 1 | 1 | **gene** | 5.52 | 13 | 452 | 4.16 | **<.0001** |  |  |  |  |  |
|  |  |  |  |  |  |  | **size*gene** | 4.66 | 13 | 452 | 3.51 | **<.0001** |  |  |  |  |  |
| H | 559 |  | **Female** | 9.035 | 1 | **0.003** | size | 0.16 | 1 | 44 | 0.75 | 0.393 |  |  |  |  |  |
|  |  |  | **Cage** | 6.840 | 1 | **0.009** | **gene** | 20.60 | 13 | 516 | 7.36 | **<.0001** |  |  |  |  |  |
|  |  |  |  |  |  |  | **size*gene** | 17.88 | 13 | 516 | 6.39 | **<.0001** |  |  |  |  |  |
|  |  |  |  |  |  |  | ***Sgk1*** |  |  |  |  |  | 0.339 | 0.074 | 182 | 4.53 | **0.0001** |
| FFh | 574 |  | **Female** | 16.648 | 1 | **<.0001** | size | 0.39 | 1 | 69 | 1.07 | 0.306 |  |  |  |  |  |
|  |  |  | Cage | 1.115 | 1 | 0.291 | **gene** | 12.94 | 13 | 533 | 2.72 | **0.001** |  |  |  |  |  |
|  |  |  |  |  |  |  | **size*gene** | 11.33 | 13 | 533 | 2.38 | **0.004** |  |  |  |  |  |

Dev.stage; developmental stage. N; total number of observations across all individuals and genes. Log expression; log2 (relative mRNA expression + 1). *X* ^2^; Chi square statistic. Chi. DF; number of degrees of freedom for the test. *P*; *P*-value of the likelihood ratio test for the random effect. Sum Sq; sum of squares. Num DF; numerator degrees of freedom. Den DF; denominator degrees of freedom based on Satterthwaite’s approximations. F; F-value. Gene slopes that remained significantly different from zero after Tukey’s adjustments are indicated, as well as slope (β), standard error (SE), degrees of freedom (DF), t-ratio (*t*) and associated *P*-values. Significant variables in bold. PF; post-fertlisation. E; eye stage. H; hatching stage. FF; first feeding stage.
